# Dendritic Mesoporous Silica-Modified Decellularized Bone Matrix Scaffold for Sustained Teriparatide Delivery in Bone Defect Repair: Characterization, Drug Release, and In Vitro Biological Performance

**DOI:** 10.3390/pharmaceutics18091067

**Published:** 2026-08-26

**Authors:** Lin Zhang, Wenbo Yang, Shipu Jia, Jing Shang, Jincheng Wang, Xin Zhao, Haotian Bai, Chenyu Wang

**Affiliations:** 1 Department of Orthopedics, The Second Hospital of Jilin University, Changchun 130041, China; zhanglin9921@mails.jlu.edu.cn (L.Z.); jiasp23@mails.jlu.edu.cn (S.J.); shangjing0811@163.com (J.S.); wangjinc@jlu.edu.cn (J.W.); 2 Department of Dermatology, The Second Hospital of Jilin University, Changchun 130041, China

**Keywords:** bone tissue engineering, drug delivery systems, decellularized bone matrix, dendritic mesoporous silica nanoparticles, teriparatide

## Abstract

**Objectives:** Critical-sized bone defects continue to represent a substantial challenge in orthopedic clinical practice. Decellularized bone matrix (DBM) possesses favorable osteoconductive properties due to its retention of native extracellular matrix architecture and collagen components. However, its limited osteogenic bioactivity restricts its application in complex bone defect repair. This study aimed to construct a dendritic mesoporous silica (DMSN)-modified DBM composite scaffold loaded with teriparatide (DBM-DMSN@TPTD) and to systematically evaluate its physicochemical properties, drug release behavior, biocompatibility, and osteogenic differentiation-promoting capacity. **Methods:** A DBM scaffold was prepared from bovine femoral cancellous bone via a combined freeze–thaw and chemical detergent decellularization method. DMSNs were synthesized through a sol–gel method, amine-functionalized with APTES, and covalently grafted onto the DBM surface via EDC/NHS crosslinking. Teriparatide was loaded onto the composite scaffolds at three concentrations (1, 10, and 100 nmol/L). The scaffolds were characterized via SEM, TEM, BET, EDS and XPS. Decellularization efficacy was assessed by DAPI staining and nucleic acid quantification. Drug release behavior was evaluated through in vitro release studies, while biocompatibility and osteogenic differentiation of rat BMSCs were examined using Live/Dead staining, phalloidin/DAPI cytoskeletal staining, CCK-8 assays, ALP staining, and RUNX2/OCN immunofluorescence. **Results:** DMSNs demonstrated a dendritic mesoporous architecture, featuring a specific surface area of 390.44 ± 1.78 m^2^/g and pore diameters within the range of 15–20 nm. DBM showed effective removal of immunogenicity, with well-preserved collagen architecture. Drug release displayed a biphasic pattern, with 56.03% released within the first 72 h and 83.23% by day 16. None of the tested scaffolds showed obvious cytotoxicity under the experimental conditions. The DBM-DMSN@TPTD-M group (10 nmol/L) produced the strongest effects on BMSC proliferation and osteogenic differentiation, as indicated by the highest ALP activity and elevated RUNX2 and OCN expression (*p* < 0.05). **Conclusions:** The DBM-DMSN@TPTD scaffold offers a native bone microenvironment, sustained drug release, and osteogenic activity in vitro. These features may support BMSC proliferation and osteogenic differentiation. Accordingly, this scaffold warrants further investigation as a potential strategy for bone defect repair.

## 1. Introduction

Bone defects are a frequent and clinically challenging problem in orthopedics, arising from congenital abnormalities, tumor resection, traumatic injury, and other causes [1]. In cases where the extent of a bone defect surpasses the crucial limit, or when it is complicated by infection or unfavorable surrounding soft tissue conditions, the body’s natural reparative capacity is often inadequate to achieve complete bone regeneration. This may consequently lead to nonunion, limb dysfunction, or even permanent disability [2]. For the repair of bone defects, the current clinical armamentarium includes autografts, allografts, metallic implants, and synthetic substitutes. Of these available options, autologous bone grafting remains the gold standard, primarily owing to its inherent triad of osteoconductive, osteoinductive, and osteogenic properties [3]. However, its clinical application is severely constrained by several associated drawbacks, including donor-site morbidity, insufficient bone supply, and the necessity of an extra operative session [4]. Although allogeneic bone grafts and metallic implants partially compensate for the insufficient availability of autologous bone, each has distinct drawbacks. Allogeneic grafts face risks of immune rejection and infection [5], while metallic implants are associated with stress shielding and suboptimal long-term osseointegration [6].

Decellularized bone matrix (DBM), which is obtained from natural bone tissue, is considered a highly promising substitute for bone grafts. This is because it retains the three-dimensional network architecture and collagen elements of the original bone extracellular matrix (ECM) [7]. Although animal-derived bone matrix is readily available, its clinical utility is constrained by host immune rejection elicited by xenograft components, including cellular debris, DNA, major histocompatibility complexes (MHC) and α-Gal epitopes [8]. Decellularization effectively eliminates immunogenic cellular components while largely preserving the native three-dimensional ultrastructure, biological activity, and specific biomechanical properties of bone, thereby offering a favorable niche that supports cell adhesion, proliferation, and osteogenic differentiation [2,9]. Bovine-derived bone substitutes are among the most widely utilized clinical options, attributed to their relatively low immunogenicity, ample supply, and well-preserved trabecular structure [10]. Numerous clinical studies have confirmed the favorable bone-healing performance of bovine-derived bone substitutes for bone defect repair [11,12]. However, in complex bone regenerative microenvironments typified by large segmental bone defects, the inherent osteoconductivity of DBM alone fails to deliver sustained osteogenic stimuli, resulting in limited bone regenerative capacity [13]. Therefore, strategies to enhance the bioactivity of natural bone matrix materials have now come to constitute a major focus in contemporary bone tissue engineering research [14,15].

A multitude of bioactive substances, including bone morphogenetic protein-2 (BMP-2), vascular endothelial growth factor (VEGF), platelet-derived growth factor (PDGF), and parathyroid hormone (PTH), have been utilized in bone tissue engineering to improve osteogenic results [16,17,18]. Among them, teriparatide (TPTD) is a bone-forming agent sanctioned by the U.S. Food and Drug Administration (FDA) for the management of osteoporosis [19], with proven clinical utility [20], a favorable safety profile [21], and demonstrated capacity to enhance osteogenesis, angiogenesis, and osseointegration [22,23,24,25]. Compared with systemic administration, the local delivery of TPTD to bone defect sites achieves higher drug concentrations at the lesion, potentiates osteogenic outcomes, and minimizes systemic exposure [22]. Canto et al. conducted a systematic review of 24 preclinical studies, confirming that local application of TPTD consistently enhances bone formation, graft integration, and angiogenesis. Shin et al. further demonstrated in an osteoporotic beagle model that local administration significantly improves bone mineral density, trabecular microarchitecture, and osseointegration outcomes [22,26]. However, as a peptide drug, TPTD is prone to rapid diffusion and degradation in vivo, necessitating the use of an appropriate drug carrier to achieve stable loading and local drug delivery [27].

Given their outstanding biocompatibility, adjustable pore dimensions, large specific surface area, and remarkable drug-carrying ability, mesoporous silica nanoparticles (MSNs) have found broad application in drug delivery and bone tissue engineering [28]. Dendritic mesoporous silica nanoparticles (DMSNs) are distinguished from conventional mesoporous materials by their unique open architecture, featuring radially oriented large pores. This structure not only enhances drug loading capacity but also provides favorable conditions for the diffusion and sustained release of macromolecular bioactive factors, making DMSNs particularly suitable for the local delivery of peptide drugs and serving as an ideal carrier for TPTD [29]. The unique chemical, physical, and structural properties of DMSNs have positioned them as versatile nanoplatforms with broad biomedical potential, spanning diagnostic bioimaging, cancer therapy, vaccine development, and drug delivery [30,31].

Based on the above considerations, we constructed a DMSN-modified DBM composite scaffold loaded with TPTD (DBM-DMSN@TPTD). In this system, DBM provides a native bone microenvironment and mechanical support, DMSN functions as a drug-delivery platform enabling efficient loading and sustained release of TPTD, and TPTD enhances local osteogenic activity. Through the synergistic action of these three components, the composite system is anticipated to promote cell adhesion and osteogenic differentiation in vitro, providing a potential bioactive scaffold design for future bone defect repair applications.

## 2. Materials and Methods

### 2.1. Preparation of Materials

#### 2.1.1. Preparation of DBM Scaffolds

Fresh bovine femoral heads (obtained from healthy cattle aged 18–24 months, immediately harvested after slaughter) were collected. After removal of soft tissues and cartilage, cancellous bone blocks were cut from the femoral heads using an oscillating saw and processed into regular rectangular specimens measuring 1.5 cm (length) × 0.3 cm (width) × 0.3 cm (height). Decellularization was conducted according to the protocol originally described by Lin et al. [32]. Briefly, the applied treatment consisted of three successive cycles of freezing and thawing, with a constant duration of 30 min for both the freezing and the thawing segments. Subsequently, the specimens were sequentially treated with 1% Triton X-100 (Sigma-Aldrich, St. Louis, MO, USA) and 1% sodium dodecyl sulfate (SDS, Sigma-Aldrich, St. Louis, MO, USA) for 24 h each, followed by incubation in DNase I solution (Sigma-Aldrich, St. Louis, MO, USA) (200 U/mL) at 37 °C for 12 h. Finally, the specimens were thoroughly rinsed with phosphate-buffered saline (PBS, Servicebio, Wuhan, Hubei, China) and preserved at −80 °C until further analysis.

#### 2.1.2. Preparation of DMSNs

The synthesis of DMSNs was conducted according to the method described by Yu et al. [33]. Briefly, cetyltrimethylammonium tosylate (CTATos, Merck KGaA, Darmstadt, Germany) (1.92 g), triethanolamine (TEAH_3_, Sinopharm Chemical Reagent Co., Ltd., Shanghai, China) (0.21 g), and 1-butyl-3-methylimidazolium trifluoromethanesulfonate ([BMIM][OTF], Sinopharm Chemical Reagent Co., Ltd., Shanghai, China) (0.02 g) were dispersed in 100 mL of deionized water, followed by magnetic stirring for 1 h at an elevated temperature of 80 °C. Subsequently, tetraethyl orthosilicate (TEOS, Sinopharm Chemical Reagent Co., Ltd., Shanghai, China) (14.58 g) was rapidly added, and the reaction was allowed to proceed at 80 °C for an additional 2 h. After the reaction, the product was pelleted by centrifugation at 10,000 rpm for 10 min. The obtained pellet was then rinsed three times using deionized water for the first two washes and anhydrous ethanol for the final wash. The obtained precipitate was dried in an oven at 100 °C overnight. To remove the template, the dried sample was calcined in a muffle furnace at 550 °C for 6 h with a heating rate of 2 °C/min. After cooling to room temperature, DMSNs were obtained.

#### 2.1.3. Amination Modification of DMSNs

To introduce amino groups onto the surface of DMSNs, the nanoparticles were modified with 3-aminopropyltriethoxysilane (APTES, Aladdin Chemistry Co., Ltd., Shanghai, China). Briefly, APTES (1.5 mL) was added to 25 mL of anhydrous ethanol and mixed by ultrasonication to form a reaction solution. The pre-synthesized DMSNs were then added to the solution, and the mixture was sealed and stirred at 80 °C for 24 h in the dark to allow APTES grafting onto the DMSNs’ surface and the inner walls of the mesoporous channels via a silanization reaction, thereby introducing amine functional groups. Once the reaction concluded, the mixed substances were left to cool spontaneously to ambient temperature. Subsequently, the resultant product was retrieved via centrifugation. To eliminate the unreacted APTES, it was then rinsed three times with absolute ethanol. Finally, the aminated DMSN (DMSN-NH_2_) was obtained by vacuum freeze-drying and stored for further use.

#### 2.1.4. Construction of DBM-DMSN Composite Scaffolds

The DBM-DMSN composite scaffolds were prepared according to a previously reported method [34]. Briefly, the lyophilized DBM scaffolds were immersed in 75% ethanol for 2 h to remove surface impurities, thoroughly rinsed with double-distilled water, and dried in a vacuum oven. The dried DBM scaffolds were then dispersed in 100 mL of ultrapure water, followed by the sequential addition of 1-ethyl-3-(3-dimethylaminopropyl) carbodiimide (EDC, Aladdin Chemistry Co., Ltd., Shanghai, China) (55 mg), N-hydroxysuccinimide (NHS, Macklin Biochemical Co., Ltd., Shanghai, China) (32.5 mg), and aminated DMSN (DMSN-NH_2_) (2 mg). The reaction mixture was stirred at 4 °C for 16 h to allow covalent grafting of DMSNs onto the collagen matrix of DBM via EDC/NHS crosslinking. After the reaction, the scaffolds were washed three times with ultrapure water, freeze-dried, and stored at −80 °C for further use.

#### 2.1.5. Preparation of Drug-Loaded Scaffolds

Teriparatide (TPTD, MedChemExpress, Shanghai, China) powder (4.12 mg) was dissolved in 1 mL of PBS to prepare a 1 mmol/L stock solution, which was then serially diluted with PBS to working solutions. The DBM-DMSN scaffolds were immersed in PBS solutions containing TPTD at the concentrations of 1, 10, and 100 nmol/L, respectively. The mixtures were sealed with parafilm, protected from light, and incubated on a horizontal shaker at 4 °C for 24 h to allow drug adsorption and loading. After loading, the scaffolds were removed, gently rinsed three times with deionized water to remove unbound drug on the surface, and lyophilized under vacuum. The resulting composite scaffolds with different drug-loading amounts were designated as DBM-DMSN@TPTD-L, DBM-DMSN@TPTD-M, and DBM-DMSN@TPTD-H.

### 2.2. Characterization of Materials

#### 2.2.1. Transmission Electron Microscopy (TEM)

Transmission electron microscopy (TEM, JEOL JEM-2100Plus, JEOL Ltd., Akishima, Tokyo, Japan) was employed to examine the internal structure, morphology, particle size, and mesoporous channel architecture of DMSNs. Images were acquired at an accelerating voltage of 200 kV. Representative images were obtained at magnifications of 50,000× and 100,000×, with scale bars of 100 and 50 nm, respectively.

#### 2.2.2. Scanning Electron Microscopy (SEM)

Scanning electron microscopy (SEM, Hitachi Regulus 8230, Hitachi High-Tech Corporation, Tokyo, Japan) was utilized to characterize the DMSNs in terms of morphology, particle size, and uniformity. Additionally, the surface morphology and homogeneity of DBM, DMSN, DBM-DMSN, and DBM-DMSN@TPTD samples were assessed using the same microscopy technique. Before SEM observation, the samples were vacuum freeze-dried, mounted on conductive carbon tape, and sputter-coated with a thin layer of gold. The elemental composition, including characteristic surface distributions of Si, O, N, and other elements, was analyzed using energy dispersive spectroscopy (EDS) equipped on the SEM. SEM images were acquired using a secondary electron detector at an accelerating voltage of 2.0 kV, a working distance of 6.1 mm, and an aperture size of 30 μm, with magnifications ranging from 500× to 20,000×. The SEM-EDS spectrum was acquired at an accelerating voltage of 15.0 kV and a magnification of 10,000×.

#### 2.2.3. X-Ray Photoelectron Spectroscopy (XPS)

X-ray photoelectron spectroscopy (K-Alpha, Thermo Fisher Scientific, Waltham, MA, USA) was employed to analyze the surface elemental composition and chemical states of DMSNs prior to and following APTES modification. Survey spectra were recorded to identify the surface elemental composition, and high-resolution N 1s spectra were acquired to evaluate the chemical state of nitrogen-containing functional groups. The atomic percentages of surface elements, especially nitrogen, were quantified to assess the degree of surface functionalization after amination.

#### 2.2.4. Mechanical Testing of DBM and DBM-DMSN Scaffolds

Mechanical testing of the DBM and DBM–DMSN scaffolds was conducted on an electronic universal testing machine (Sinter, Shanghai, China). The load cell was zeroed prior to testing, and each specimen was mounted vertically at the center of the compression platen. A constant displacement rate of 0.1 mm/min was applied until a stable stress–strain curve was obtained. The compressive strength corresponded to the maximum stress on the curve, whereas the elastic modulus was calculated from the slope of the initial linear portion. For each scaffold type, three independently prepared scaffold specimens were tested (*n* = 3).

#### 2.2.5. Nitrogen Adsorption–Desorption Analysis

The specific surface area and pore characteristics of DMSN samples were determined using nitrogen adsorption–desorption measurements (Micromeritics ASAP 2460, Norcross, GA, USA). The Brunauer–Emmett–Teller (BET) method was used to calculate the specific surface area, and the Barrett–Joyner–Halenda (BJH) method was applied to determine the pore size distribution. Prior to analysis, the samples were degassed at 100 °C for 12 h, and measurements were performed at 77 K.

#### 2.2.6. Characterization of DBM Decellularization

Prepared DBM scaffolds were decalcified, embedded in paraffin, and sectioned into slices, followed by hematoxylin and eosin (H&E) staining for histological assessment. Additionally, fresh bone tissue (FBT) and DBM sections were stained with DAPI (Sigma-Aldrich, St. Louis, MO, USA) to observe residual cell nuclei under a fluorescence microscope. DNA and RNA contents were quantified using a DNA/RNA extraction kit (Beijing Genenode Biotech Co., Ltd., Beijing, China) and a UV spectrophotometer (BioSpectrometer, Eppendorf SE, Hamburg, Germany).

### 2.3. Isolation and Culture of Rat Bone Marrow Mesenchymal Stem Cells (BMSCs)

All procedures involving rats received ethical approval (approval no. 2024-555). Under aseptic conditions, BMSCs were extracted from the femurs and tibias of Sprague–Dawley rats. Once the epiphyses were opened, the bone marrow cavity was irrigated with Dulbecco’s Modified Eagle’s Medium/F-12 (DMEM/F-12, Gibco, Grand Island, NY, USA). This medium was enriched with 10% fetal bovine serum (FBS) (EvaCell, Suzhou, Jiangsu, China) and 1% penicillin-streptomycin (EvaCell, Suzhou, Jiangsu, China). The gathered cell suspension was then passed through a 200-mesh cell filter. Subsequently, it was inoculated into culture dishes at a concentration of 2 × 10^5^ cells per square centimeter. The cells were cultivated in a CO_2_ incubator (BB 150, Thermo Fisher Scientific, Waltham, MA, USA) maintained at 37 °C with a 5% CO_2_ atmosphere. The culture medium was refreshed every two days. When the cells achieved 90% confluence, they were treated with 0.25% trypsin (Gibco, Grand Island, NY, USA) for digestion and then sub-cultured. For all in vitro experiments, BMSCs at the third passage (P3) were employed.

### 2.4. Biocompatibility Evaluation of Composite Scaffolds

#### 2.4.1. CCK-8 Cell Proliferation Assay

The scaffolds of each group were sterilized via gamma irradiation and placed into 24-well plates. BMSCs were then applied to the scaffolds at a density of 5 × 10^3^ cells per construct, with three replicates per group (*n* = 3). Following culture at 37 °C with 5% CO_2_ for 1, 4, and 7 days, cell proliferation was quantified with the Cell Counting Kit-8 (CCK-8) (Biossynthesis Biotechnology Co., Ltd., Beijing, China). Subsequently, the optical density (OD) was measured at 450 nm using a microplate reader (MULTISKAN MK3, Thermo Fisher Scientific, Waltham, MA, USA).

#### 2.4.2. Live/Dead Staining

BMSCs were seeded in 24-well plates onto the scaffolds at 2 × 10^4^ cells per well. Following a 3-day culture period at 37 °C, the supernatant was removed, and the cells were washed twice with PBS. Then, 300 μL of working solution containing AM and PI (Calcein-AM/PI double staining kit, BestBio, Shanghai, China) was added to each well and incubated at 37 °C in the dark for 30 min. After staining, the cells were washed three times with PBS to remove residual dye. Fluorescence images were captured using an inverted fluorescence microscope (ECHO REVOLVE, San Diego, CA, USA), with green fluorescence indicating live cells and red fluorescence representing dead cells. Images were analyzed using ImageJ (version 2.14.0, National Institutes of Health, Bethesda, MD, USA) under identical image-acquisition and analysis settings. The percentage of live cells was quantified from the Live/Dead images.

#### 2.4.3. Cytoskeletal and Nuclear Staining

To observe the adhesion and spreading morphology of BMSCs on the surface of each scaffold group, BMSCs were seeded onto the scaffolds in 24-well plates at a density of 2 × 10^4^ cells per well, with three replicates per group (*n* = 3). After 3 days of culture, the cells were fixed with 4% paraformaldehyde (Servicebio, Wuhan, Hubei, China) for 20 min and permeabilized with 0.1% Triton X-100 for 10 min. Subsequently, the cells were stained with 5 μg/mL Rhodamine-phalloidin (Thermo Fisher Scientific, Waltham, MA, USA) at room temperature in the dark for 30 min. After washing with PBS, the nuclei were counterstained with DAPI for 5 min. After staining, the samples were washed with PBS, mounted in anti-fade medium on glass slides, and examined using an inverted fluorescence microscope.

### 2.5. Evaluation of Osteogenic Differentiation

Alkaline Phosphatase (ALP) Staining: BMSCs were seeded at a density of 2 × 10^4^ cells per well into 24-well plates containing the scaffolds, with three replicates per group. Osteogenic induction medium (Xirui infinity Biotechnology Co., Ltd., Hangzhou, Zhejiang, China) was used to induce osteogenic differentiation. After osteogenic induction for 7 days at 37 °C with 5% CO_2_, the cells were fixed with 4% paraformaldehyde at room temperature for 15 min. Following PBS washing, the staining working solution was prepared using a BCIP/NBT Alkaline Phosphatase Color Development Kit (Beyotime Biotechnology, Shanghai, China) and added to each well, followed by incubation at room temperature in the dark for 30 min. After color development, the staining solution was discarded, and the reaction was terminated by repeated rinsing with distilled water. Images were captured under an inverted light microscope (ECHO REVOLVE, San Diego, CA, USA), with blue-violet positive staining areas indicating ALP-positive expression.

Immunofluorescence Staining: BMSCs were seeded at a concentration of 1 × 10^4^ cells per well into 24-well plates that held the scaffolds. After a week of co-culture with the scaffolds under osteogenic induction conditions, the scaffolds were removed. The cells situated at the base of the plate were fixed with 4% paraformaldehyde at room temperature for 20 min, washed with PBS, and permeabilized with 0.1% Triton X-100 for 10 min. Subsequently, the cells were blocked with 5% bovine serum albumin (BSA, Sigma-Aldrich, St. Louis, MO, USA) at 37 °C for 30 min. Primary antibodies against RUNX2 (ABclonal Technology Co., Ltd., Wuhan, Hubei, China) and OCN (ABclonal Technology Co., Ltd., Wuhan, Hubei, China) were added and incubated overnight at 4 °C in a humidified chamber. The next day, the cells were washed three times with PBS and then incubated with Alexa Fluor 594-conjugated secondary antibody (ABclonal Technology Co., Ltd., Wuhan, Hubei, China) for 1 h at room temperature in the dark. After three washes with PBS, the nuclei were counterstained with DAPI for 5 min. Following staining, the cells were immediately observed and imaged under an inverted fluorescence microscope, with RUNX2 and OCN appearing red and nuclei blue. The mean fluorescence intensities of RUNX2 and OCN were measured using ImageJ (version 2.14.0, National Institutes of Health, Bethesda, MD, USA) under identical image-acquisition and analysis settings. Three independent biological replicates were analyzed for each group (*n* = 3).

### 2.6. In Vitro Drug Release

To determine the drug loading capacity of the scaffolds, DBM and DBM-DMSN scaffolds were separately immersed in TPTD solution (10 nmol/L) at 4 °C for 24 h under gentle shaking. After loading, the scaffolds were removed, and the residual drug concentration in the supernatant was measured using a PTH(1–34) ELISA kit (Boyan Biosciences, Shanghai, China). The encapsulation efficiency (EE%) was derived from the following expression:

$$EE\left(\%\right)=\frac{\text{Weight}\;\text{of}\;\text{encapsulated}\;\text{TPTD}\times 100\%}{\text{Weight}\;\text{of}\;\text{initial}\;\text{TPTD}}$$

For the release study, the TPTD-loaded DBM and DBM-DMSN scaffolds were separately immersed in centrifuge tubes containing 10 mL of PBS (pH 7.4) and incubated at 37 °C in a shaker at 100 rpm. At each sampling time, 1 mL of the supernatant was withdrawn, and the same amount of fresh PBS was added. The drug concentration in the collected samples was determined using the same ELISA kit, and the cumulative release profile was plotted. All experiments were performed in triplicate.

### 2.7. Statistical Analysis

All experimental data are presented as mean ± standard deviation (SD), with at least three independent replicates per group. For cell-based experiments, *n* = 3 represents three independent biological replicates. For material characterization, mechanical testing, and drug-loading/release experiments, *n* = 3 represents independently prepared samples or specimens, as specified in the corresponding sections. Statistical evaluations were carried out employing the GraphPad Prism 8.0 software (GraphPad Software, Boston, MA, USA). Comparisons between two groups were conducted using independent-sample *t*-tests, while comparisons among multiple groups were performed using one-way analysis of variance (ANOVA) followed by Tukey’s multiple comparisons post hoc test. Because each group contained three biological replicates, formal normality testing has limited statistical power; therefore, data distribution and statistical results were interpreted cautiously. A *p*-value of <0.05 was considered statistically significant, with significance levels denoted as * *p* < 0.05, ** *p* < 0.01, and *** *p* < 0.001.

## 3. Results and Discussion

### 3.1. DMSN and DBM Were Successfully Prepared

**Figure 1:**
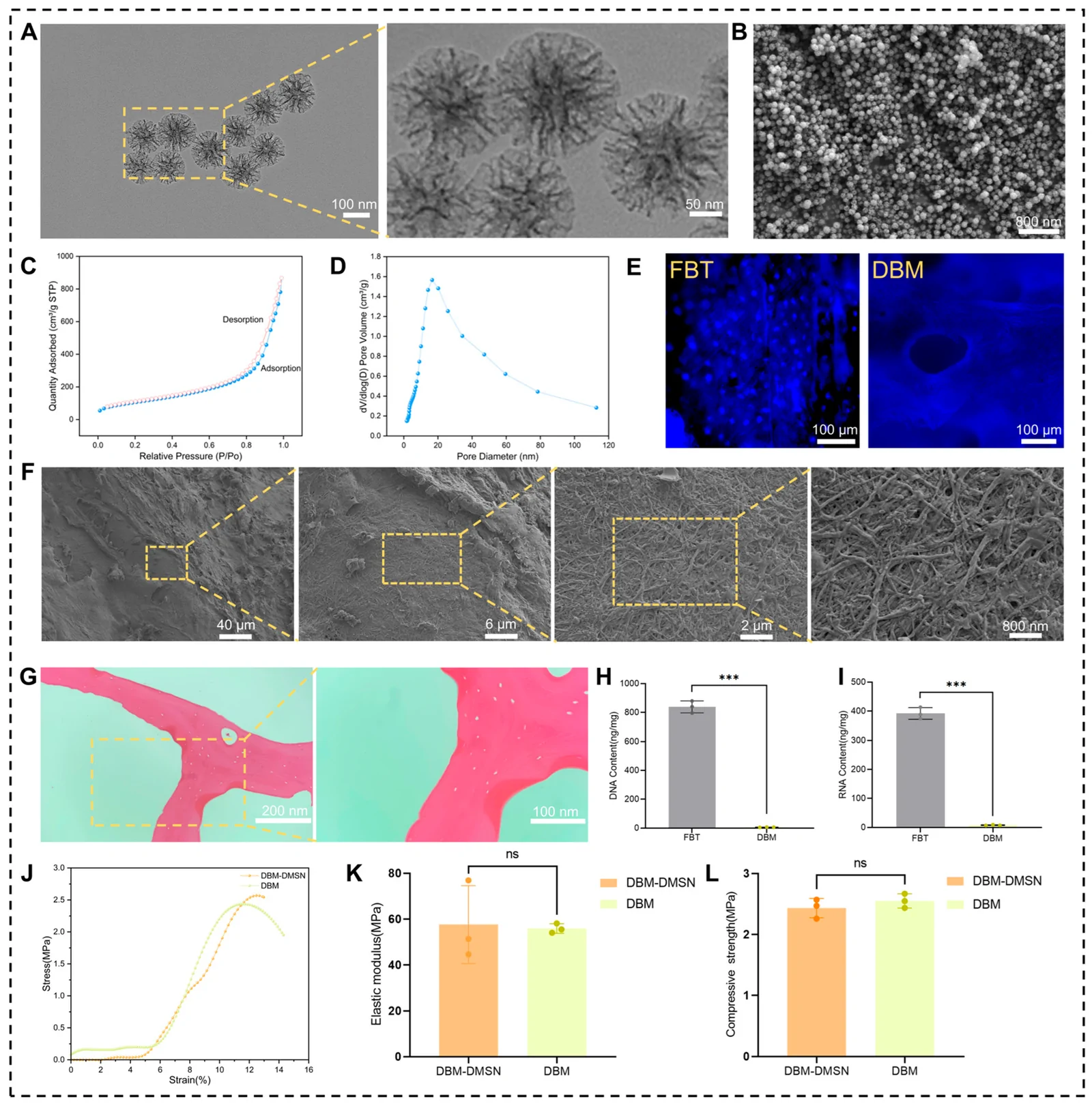
Preparation and characterization of DBM and DMSN. (**A**) TEM image of DMSNs. (**B**) SEM image of DMSNs. (**C**) N_2_ adsorption–desorption isotherm of DMSNs. (**D**) Pore size distribution curve of DMSNs. (**E**) DAPI staining of FBT and DBM. (**F**) SEM image of DBM showing the collagen fiber network. (**G**) H&E staining of DBM. (**H**,**I**) Quantitative analysis of DNA and RNA content in FBT and DBM. (**J**) Representative stress–strain curves of DBM and DBM-DMSN scaffolds. (**K**) Quantitative analysis of elastic modulus. (**L**) Quantitative analysis of compressive strength. Data are presented as mean ± SD (*n* = 3). Comparisons between two groups were performed using independent-sample *t*-tests. ns, no significant difference; *** *p* < 0.001.

In this study, DMSNs with a dendritic mesoporous structure, high specific surface area, and large pore size were successfully synthesized, and DBM scaffolds with preserved extracellular matrix architecture were prepared via decellularization. TEM images showed that the synthesized DMSNs were approximately 100 nm in diameter and displayed a uniform spherical shape with a relatively narrow size distribution. Internally, they possessed a typical radially dendritic mesoporous structure, with mesopore channels radiating outward from the particle center to form an open three-dimensional interconnected pore network, facilitating effective therapeutic-molecule loading and sustained release (Figure 1A). SEM images further confirmed the good dispersibility and uniform spherical morphology of DMSN (Figure 1B). The nitrogen adsorption–desorption isotherms displayed a typical type IV curve with a distinct H3-type hysteresis loop (Figure 1C). This suggests the existence of a large number of mesoporous structures. BET analysis revealed a specific surface area of 390.44 ± 1.78 m^2^/g and a total pore volume of 1.32 cm^3^/g. BJH analysis showed adsorption and desorption average pore diameters of 13.26 nm and 12.74 nm, respectively. The pore size distribution was mainly concentrated in the range of 15–20 nm (Figure 1D). These large pore size and pore volume parameters provided a structural foundation for the efficient loading and sustained release of TPTD. In parallel, DBM scaffolds were further prepared and characterized by multiple methods to assess the decellularization outcome. DAPI staining revealed that, in contrast to fresh bone tissue (FBT), which exhibited numerous blue-fluorescent nuclei, the decellularized DBM showed almost no residual nuclear signals (Figure 1E), indicating effective removal of cellular components. DNA and RNA quantification further supported this conclusion. The DNA content decreased from 837.83 ± 41.77 ng/mg in fresh bone tissue to 6.22 ± 1.13 ng/mg after decellularization, while the RNA content decreased from 391.75 ± 20.12 ng/mg to 7.94 ± 1.41 ng/mg. Both DNA and RNA contents were significantly reduced in the decellularized samples compared with fresh bone tissue (*p* < 0.001; Figure 1H,I), confirming the effective removal of residual nucleic acids. SEM observation revealed that the decellularization process not only effectively removed cellular components but also adequately exposed the well-organized collagen fiber network within the bone matrix (Figure 1F), while H&E staining further confirmed that the collagen structure and distribution were well preserved (Figure 1G). To further evaluate whether DMSN modification affected the mechanical properties of the DBM scaffold, uniaxial compression testing was performed. The stress–strain curves showed similar mechanical behavior between the DBM and DBM-DMSN scaffolds (Figure 1J). The elastic modulus values were 55.91 ± 2.06 MPa for DBM and 57.64 ± 17.03 MPa for DBM-DMSN (Figure 1K), while the compressive strength values were 2.55 ± 0.12 MPa for DBM and 2.43 ± 0.16 MPa for DBM-DMSN, respectively (Figure 1L). No significant differences were observed in either compressive strength or elastic modulus between the two groups, suggesting that DMSN modification did not markedly compromise the mechanical properties of the DBM scaffold. Collectively, these results demonstrate the successful fabrication of DBM scaffolds with an intact native extracellular matrix architecture and significantly reduced immunogenicity.

Decellularization effectively eliminates immunogenic cellular components while largely preserving the three-dimensional ultrastructure and bioactivity of native bone tissue, thereby providing a favorable microenvironment for cell adhesion, proliferation, and osteogenic differentiation. Previous studies have demonstrated that DNA residue in decellularized matrices is a critical indicator for evaluating immunogenicity, and it is generally accepted that residual DNA below 50 ng/mg dry weight is considered indicative of thorough decellularization [35]. Notably, the design of decellularization strategies requires a delicate balance between removing immunogenic components and preserving ECM integrity. Freeze–thaw pretreatment effectively lyses cells, and subsequent treatment with appropriate concentrations of chemical detergents further removes cellular debris. However, excessive treatment may compromise collagen structure and bioactive components such as glycosaminoglycans [36]. In the present study, the well-preserved collagen fiber network indicates that the adopted decellularization protocol effectively reduces immunogenicity. At the same time, it maximally preserves the inherent microstructure of bone tissue. As a result, it offers a conducive biological microenvironment that promotes cell adhesion, proliferation, and osteogenic differentiation.

While DBM provides structural support and a favorable microenvironment, its intrinsic osteoinductive activity is limited and insufficient to drive sustained bone regeneration [13]. To address this limitation, we introduced DMSN as a drug delivery platform. Compared with conventional mesoporous silica, DMSN possesses a unique radially open pore architecture, larger pore volume, and higher drug-loading capacity [29,37,38], and can also protect drugs from enzymatic degradation [39,40]. Kassem et al. employed amine-functionalized DMSN to load simvastatin, achieving a drug loading capacity exceeding 20% and demonstrating sustained release via Fickian diffusion, which further highlights the structural advantages of DMSN for sustained drug delivery [41]. Therefore, the introduction of DMSN established a structural foundation for the efficient loading and sustained release of TPTD.

### 3.2. Characterization of the DBM-DMSN Composite Scaffold

Amination modification of DMSN is the key to achieving covalent grafting onto the DBM scaffold. To verify the success of amination modification on the DMSN surface, X-ray photoelectron spectroscopy (XPS) was employed to analyze the surface elements and chemical states of DMSNs before and after amination.

**Figure 2:**
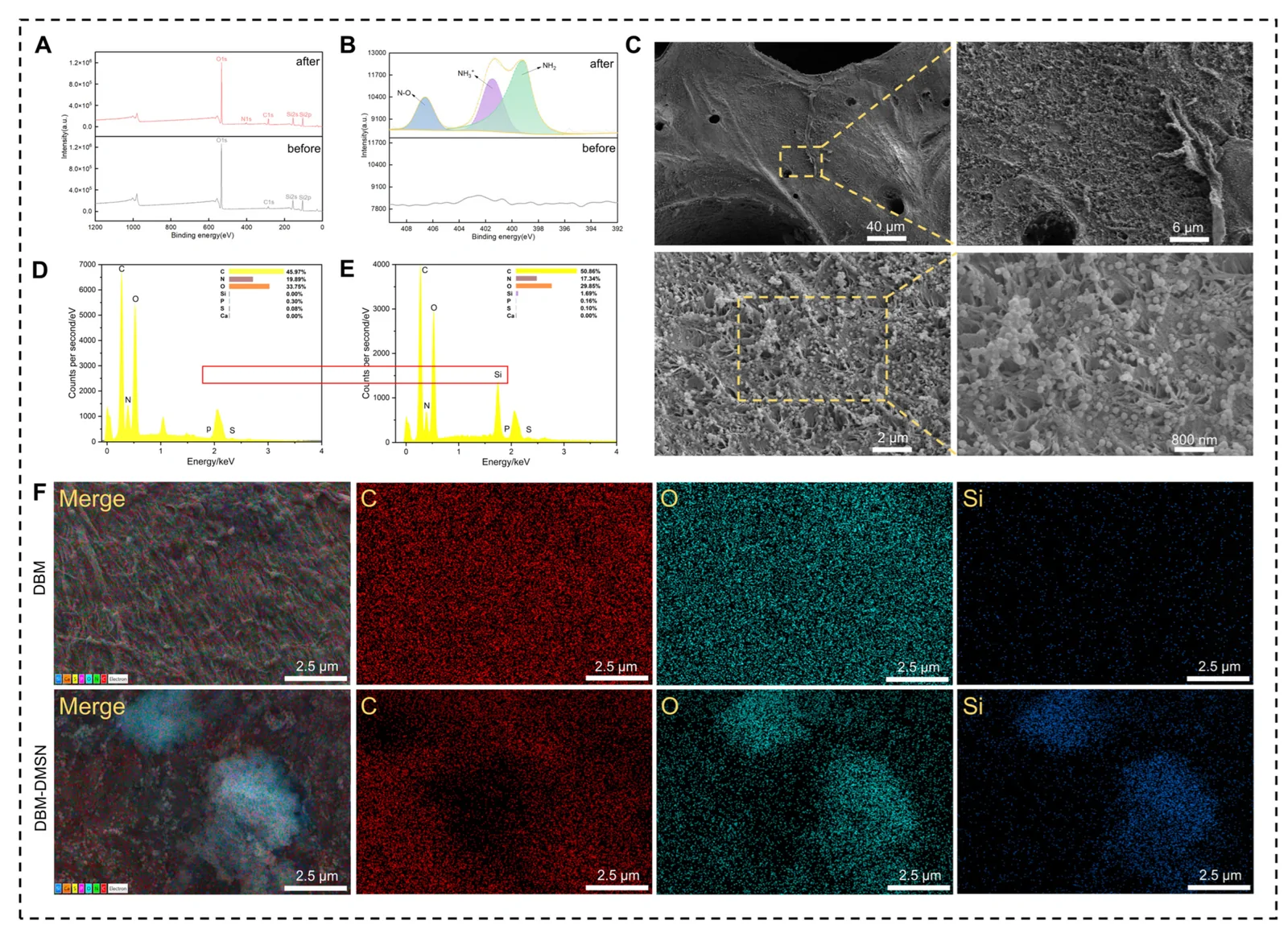
Characterization of DMSN-NH_2_ and DBM-DMSN. (**A**) XPS survey spectra of DMSN before and after APTES modification. (**B**) High-resolution N 1s XPS spectrum of APTES-modified DMSN. (**C**) SEM images of DBM-DMSN. (**D**) EDS spectra of DBM and (**E**) DBM-DMSN. (**F**) EDS elemental mapping of DBM-DMSN.

The survey spectra showed that the unmodified DMSN mainly exhibited characteristic signals of Si 2p (~103 eV), Si 2s (~154 eV), and O 1s (~532 eV), consistent with the chemical composition of silica. After APTES modification, a distinct N 1s signal peak (~400 eV) clearly appeared (Figure 2A), indicating the successful introduction of amino groups (-NH_2_) onto the DMSN surface. Quantitative XPS elemental analysis further showed that the nitrogen atomic percentage increased from 0.70 at% in unmodified DMSN to 3.89 at% in APTES-modified DMSN, providing quantitative evidence for nitrogen-containing surface functionalization. Further high-resolution N 1s spectra fitting revealed (Figure 2B) that the peak could be deconvoluted into two components. The peak at 399.4 eV was assigned to free amino groups (-NH_2_). The shoulder at 401.5 eV was attributed to protonated amine groups (-NH_3_^+^) or to amino groups forming hydrogen bonds with silanol groups (Si-OH) on the DMSN surface, reflecting the interaction between the grafted amines and the silica support. These XPS results confirm the successful introduction of amino groups on the DMSN surface, enabling TPTD loading via ionic interactions.

SEM images revealed that the unmodified DBM surface exhibited a loosely arranged collagen fiber network with visible inter-fiber pores; after DMSN modification, numerous spherical nanoparticles were uniformly distributed on the scaffold surface and within the collagen fiber interspaces, indicating successful binding of DMSN to the DBM scaffold (Figure 2C). EDS analysis showed that the DBM group mainly contained C, N, and O elements (Figure 2D). This finding aligns with the chemical makeup of the bone matrix, where collagen serves as the principal organic constituent, while the DBM-DMSN group exhibited a distinct Si characteristic peak under the same detection conditions (Figure 2E), confirming the successful coating of DMSN (with SiO_2_ as the main component). Further elemental mapping clearly showed that Si elements were uniformly distributed on the scaffold surface (Figure 2F), indicating that DMSNs could be stably and uniformly anchored across the entire surface area of the DBM scaffold. Collectively, these results confirmed the successful construction of the DBM-DMSN composite scaffold, providing an ideal delivery platform for subsequent drug loading and sustained release.

EDC/NHS crosslinking is a widely used zero-length crosslinking strategy in the biomaterials field [42,43]. The process proceeds through EDC-mediated activation of carboxyl groups, followed by NHS conversion of the resulting intermediate into a more stable NHS ester that subsequently reacts with amino groups to produce amide bonds [44]. In this study, the EDC/NHS system activated carboxyl groups on glutamic and aspartic acid residues of collagen fibers in DBM, forming stable amide bonds with primary amine groups on the DMSN-NH_2_ surface, thereby achieving covalent immobilization of DMSNs onto the DBM surface. Amination modification is a critical prerequisite for achieving covalent grafting of DMSNs onto DBM. Hartono et al. confirmed the successful grafting of APTES onto mesoporous silica surfaces using multiple techniques including XPS, solid-state NMR, and TGA, and found that the protein adsorption capacity of the aminated material increased by 8-fold [45]. This enhancement in protein affinity is particularly favorable for the loading of peptide drugs such as TPTD.

### 3.3. Evaluation of TPTD Loading and Release Performance

**Figure 3:**
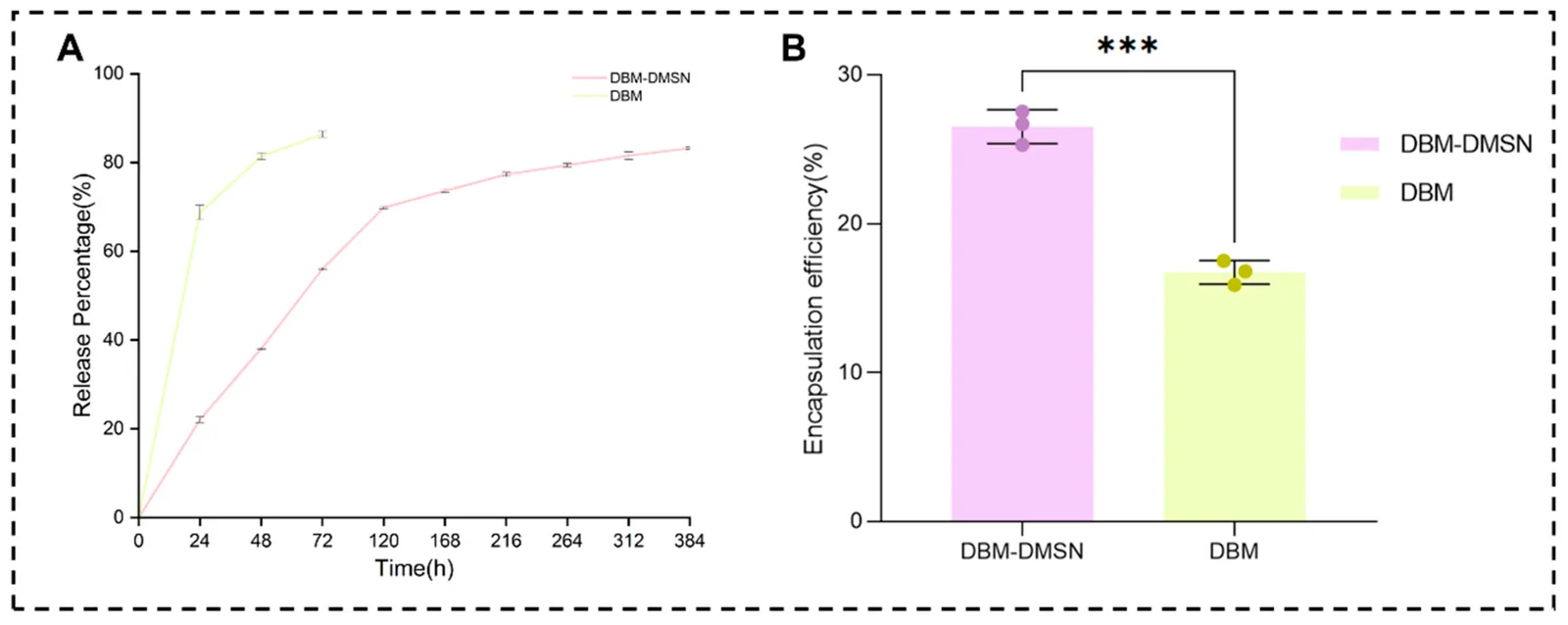
TPTD loading and release behavior of DBM-DMSN and DBM scaffolds. (**A**) In vitro cumulative release profiles of TPTD from DBM-DMSN and DBM scaffolds. (**B**) Encapsulation efficiency of TPTD in DBM-DMSN and DBM scaffolds. Data are presented as mean ± SD (*n* = 3). Comparisons between two groups were performed using independent-sample *t*-tests. *** *p* < 0.001.

Based on the successful construction of the DBM-DMSN composite scaffold, TPTD was further loaded onto the scaffold, and its in vitro loading and release behavior was systematically evaluated. To clarify the contribution of DMSN modification to TPTD retention and sustained release, TPTD-loaded DBM without DMSN modification was included as a comparison group under the same loading condition. The encapsulation efficiency results (Figure 3B) showed that the encapsulation efficiency of TPTD in the DBM-DMSN scaffold was 26.51% ± 1.14%, which was markedly greater than the value obtained for the DBM scaffold without DMSN modification (16.73% ± 0.81%, *p* < 0.001).

The in vitro release results (Figure 3A) showed that both scaffolds exhibited an initial phase of swift release followed by a phase of slower, continuous release. However, the scaffold without DMSN modification showed a faster release profile, with cumulative TPTD release reaching 68.77% ± 1.63% at 24 h and 86.37% ± 0.72% at 72 h. In contrast, the DBM-DMSN@TPTD scaffold exhibited a more sustained release profile, with cumulative release reaching 56.03% ± 0.26% during the initial 72 h and gradually increasing to 83.23% ± 0.25% by day 16, after which the release curve gradually plateaued.

To further interpret the release behavior, the early-stage release data were fitted using the Korsmeyer–Peppas model, a classical empirical model for porous or matrix-based delivery systems [46]. The fitting showed a high correlation with an *R*^2^ value of 0.9943 and a release exponent n of 0.87, indicating that the early-stage TPTD release involved anomalous transport rather than simple diffusion alone. The biphasic release profile, characterized by an initial burst and a subsequent sustained phase, may enable rapid establishment of effective drug concentrations during the initial phase of bone defect repair. This is followed by prolonged osteogenic stimulation, which may provide sustained support for bone healing over an extended period.

The improved TPTD loading and sustained release observed in the DMSN-modified scaffold may be attributed to the structural and chemical features of DMSN. Due to its substantial pore volume, elevated specific surface area, and open mesoporous structure, DMSN has been extensively employed for the loading and prolonged release of biomolecules [38,47]. Moreover, potential non-covalent associations, like electrostatic forces and hydrogen bonds, that can occur between TPTD and the aminated surface of DMSN might also play a role in drug retention [48]. Meanwhile, because DBM is mainly composed of collagen-based extracellular matrix components, non-covalent adsorption of TPTD onto the DBM matrix may also participate in TPTD retention [49]. However, since TPTD was loaded after the EDC/NHS-mediated construction of the DBM-DMSN scaffold, covalent binding between TPTD and the DBM matrix is unlikely to be the dominant loading mechanism.

Consistently, the Korsmeyer–Peppas fitting result further supported the biphasic release behavior of TPTD from the DBM-DMSN scaffold. Under the commonly used cylindrical-matrix approximation, n values between approximately 0.45 and 0.89 are generally associated with anomalous transport, whereas an n value close to 0.89 approaches Case II transport [50]. The n value of 0.87 therefore suggests that diffusion contributed substantially to TPTD release, while matrix relaxation or structural changes and possible drug–matrix interactions may also have played important roles. Because the DBM-DMSN scaffold has a heterogeneous porous structure rather than an ideal regular geometry, this interpretation should be considered approximate. Overall, the fitting results suggest that TPTD release was governed by multiple concurrent processes rather than by a single diffusion-controlled mechanism. This biphasic release pattern can be advantageous for bone repair. The initial release stage might offer prompt osteoinductive stimulation. Subsequently, the continuous release stage can assist in keeping the local area exposed to the peptide during the processes of osteogenic differentiation and matrix maturation [51]. The potential relevance of this release profile is supported by previous preclinical studies of local PTH(1–34) delivery. In a rat critical-sized bone defect model, PTH(1–34) released from a hydrogel-based scaffold remained biologically active for up to 21 days and improved defect bridging compared with the scaffold without PTH(1–34) [52]. However, previous studies comparing different PTH release patterns suggest that the biological response may depend on the temporal profile of exposure, and prolonged continuous release cannot automatically be considered equivalent to intermittent or pulsatile administration [53]. In the present study, the biphasic release profile and approximately 83% cumulative release over 16 days indicate the potential of the DBM-DMSN scaffold to prolong local TPTD availability. Nevertheless, these in vitro data do not establish the therapeutic window, optimal exposure pattern, or in vivo pharmacodynamic efficacy of the released TPTD. Further studies involving local drug-concentration monitoring, in vivo pharmacokinetic and pharmacodynamic analyses, and direct comparison with single-dose or intermittent administration are therefore required.

### 3.4. DBM-DMSN@TPTD Composite Scaffold Exhibits Good Biocompatibility

**Figure 4:**
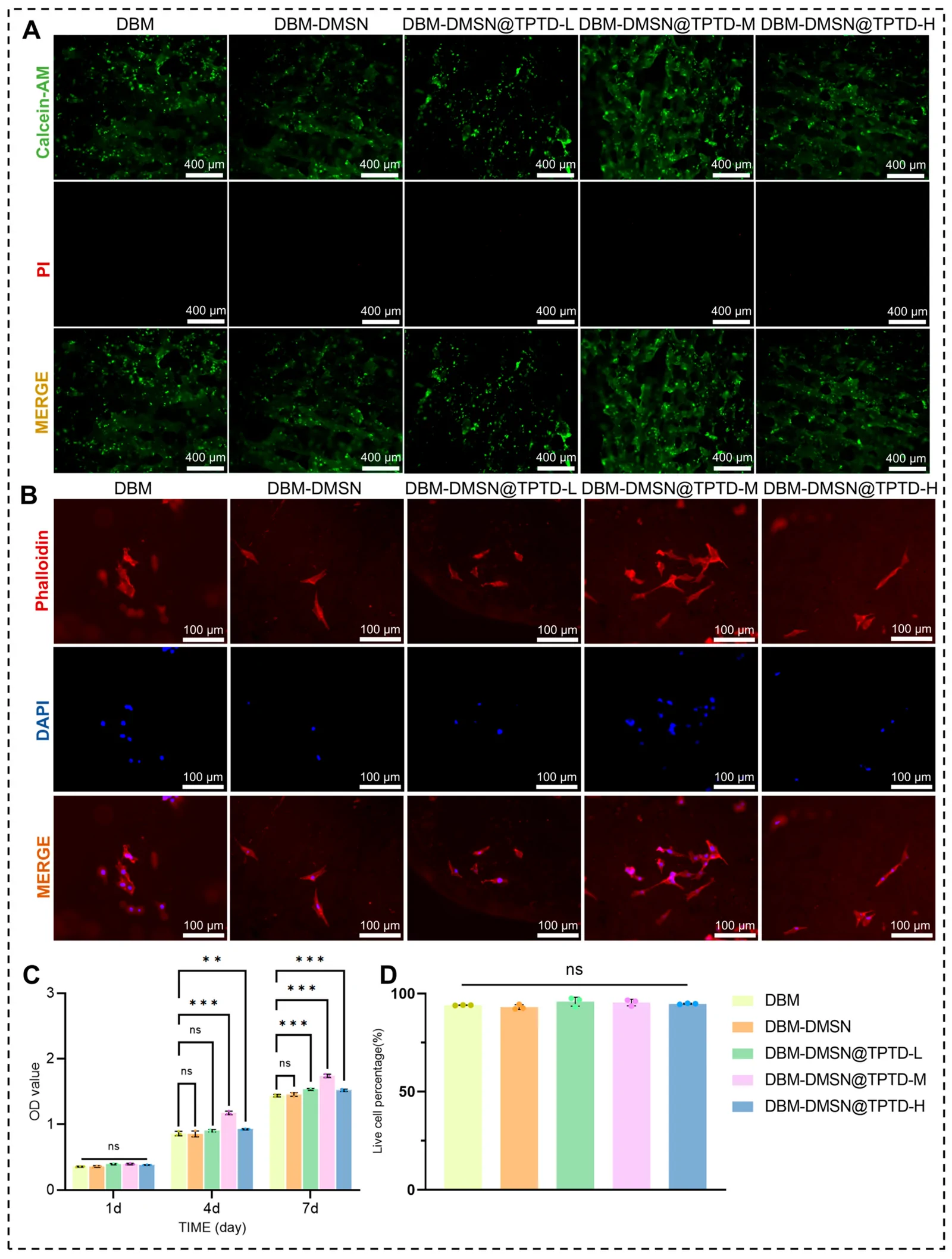
Biocompatibility evaluation of DBM-DMSN@TPTD composite scaffold. (**A**) Live/Dead staining of BMSCs cultured on different scaffolds (green: live cells; red: dead cells). (**B**) Phalloidin/DAPI staining of BMSCs showing cytoskeletal organization and cell morphology (red: F-actin; blue: nuclei). (**C**) CCK-8 assay of BMSCs cultured on different scaffolds for 1, 4, and 7 days. (**D**) Quantitative analysis of live cell percentage based on Live/Dead staining. Data are presented as mean ± SD (*n* = 3). Comparisons among multiple groups were performed using one-way ANOVA followed by Tukey’s multiple comparisons post hoc test. ns, no significant difference; ** *p* < 0.01; *** *p* < 0.001.

Excellent biocompatibility serves as a crucial requirement for the clinical utilization of bone repair scaffolds. Live/Dead staining results (Figure 4A) showed that a large number of green-fluorescent live cells were observed on the surface of all scaffold groups, whereas only a few red-fluorescent dead cells were observed, suggesting that none of the scaffolds exhibited obvious cytotoxicity and that cell viability remained at a high level. Quantitative analysis of Live/Dead staining further showed that the live cell percentage remained high in all groups, with no significant differences among the groups (Figure 4D), confirming the good cytocompatibility of the scaffolds. DBM has been confirmed to possess good biocompatibility and low immunogenicity, consistent with previous reports. Its decellularization treatment effectively removes immunogenic cellular components, while preserving the three-dimensional structure and collagen components of the native ECM [2,9].

Phalloidin/DAPI cytoskeletal staining results (Figure 4B) further revealed that BMSCs showed good attachment and spreading across all scaffold surfaces, displaying a typical elongated spindle-shaped morphology. Notably, the TPTD-loaded groups (especially the DBM-DMSN@TPTD-M group) exhibited better cell spreading compared with the DBM-DMSN and DBM groups, with more abundant pseudopodia and clearer and more intact actin cytoskeletons, suggesting that the release of TPTD may facilitate cytoskeletal remodeling and cell spreading.

CCK-8 proliferation assay results (Figure 4C) showed that cell proliferation activity in all groups increased in a time-dependent manner with prolonged culture time (1, 4, and 7 days). At all time points, the cell proliferation activity of the DBM-DMSN group was comparable to that of the DBM group (*p* > 0.05), indicating that DMSN modification did not introduce obvious cytotoxicity to the scaffold system, which is consistent with the well-documented biosafety of DMSN in the literature [38].

Sun et al. demonstrated that DMSN exhibits excellent biocompatibility and biosafety both in vitro and in vivo when used for vaccine delivery, with favorable degradability [54]. Zhu et al. also confirmed the good biocompatibility and stability of DMSN through an MTT assay and hemolysis test [55]. Among the TPTD-loaded groups, the DBM-DMSN@TPTD-M group exhibited significantly higher cell proliferation activity than the DBM and DBM-DMSN groups at days 4 and 7 (*p* < 0.05), while the DBM-DMSN@TPTD-H group showed higher proliferation activity than the unloaded controls but slightly lower than the medium-dose group. These findings suggest that TPTD-loaded composite scaffolds at appropriate concentrations possess good cytocompatibility and can promote BMSC proliferation. The enhanced BMSC proliferation observed in the TPTD-loaded groups, especially the medium-dose group, is consistent with the known biological effects of TPTD. Previous studies have reported that PTH/PTH(1–34) may regulate BMSC proliferation and fracture-healing-related cellular responses through signaling pathways involving cAMP/PKA/CREB [56]. In addition, the biological effects of TPTD may depend on both exposure time and concentration [57]. Thus, the enhanced proliferation observed in the TPTD-loaded groups may be partly associated with the signaling responses reported for PTH(1–34), although the precise molecular events responsible for this effect remain to be clarified.

### 3.5. DBM-DMSN@TPTD Composite Scaffold Promotes Osteogenic Differentiation of BMSCs

**Figure 5:**
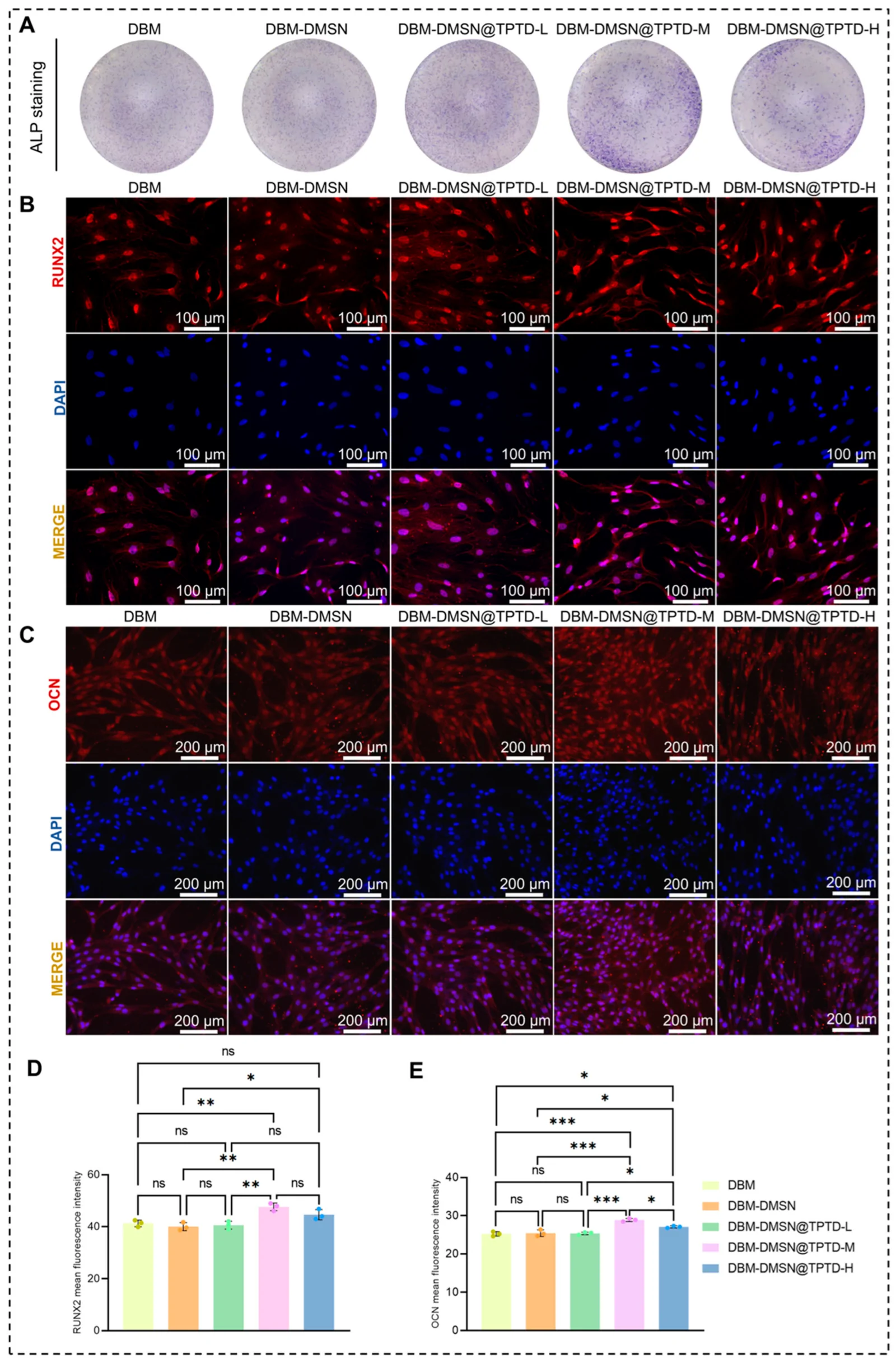
Osteogenic differentiation-promoting effect of DBM-DMSN@TPTD composite scaffold on BMSCs. (**A**) ALP staining of BMSCs after 7 days of culture. (**B**) Immunofluorescence staining of RUNX2 in BMSCs. (**C**) Immunofluorescence staining of OCN in BMSCs. (**D**) Quantitative analysis of RUNX2 mean fluorescence intensity. (**E**) Quantitative analysis of OCN mean fluorescence intensity. Data are presented as mean ± SD (*n* = 3). Comparisons among multiple groups were performed using one-way ANOVA followed by Tukey’s multiple comparisons post hoc test. ns, no significant difference; * *p* < 0.05; ** *p* < 0.01; *** *p* < 0.001.

ALP staining results (Figure 5A) indicated that the DBM-DMSN@TPTD-M group exhibited the deepest blue-violet staining and the largest positive area among all groups, indicating the most robust ALP activity. In contrast, the DBM-DMSN@TPTD-H group, despite showing significantly better osteogenic indicators than the unloaded control group, did not exhibit further advantages over the M group, suggesting that increasing the TPTD concentration from 10 nmol/L to 100 nmol/L did not yield additional osteogenic benefits.

RUNX2 immunofluorescence staining results (Figure 5B) showed that all groups exhibited some degree of RUNX2-positive expression, with stronger fluorescence signals observed in the DBM-DMSN@TPTD-M and DBM-DMSN@TPTD-H groups. Semi-quantitative assessment of RUNX2 mean fluorescence intensity further confirmed this trend (Figure 5D). The DBM-DMSN@TPTD-M group exhibited the highest RUNX2 fluorescence intensity and was significantly higher than the DBM and DBM-DMSN groups, while the DBM-DMSN@TPTD-H group also showed an increased RUNX2 fluorescence intensity compared with the DBM-DMSN group. These results suggest that the medium dose of TPTD produced the most pronounced osteogenic differentiation-related response among the tested concentrations.

OCN immunofluorescence staining results (Figure 5C) showed a trend generally consistent with RUNX2, with the most prominent fluorescence signal observed in the DBM-DMSN@TPTD-M group. Semi-quantitative analysis of OCN mean fluorescence intensity showed that the DBM-DMSN@TPTD-M group had the highest OCN expression, while the DBM-DMSN@TPTD-H group showed a moderate increase (Figure 5E). In contrast, the low-dose group did not show an obvious increase compared with the DBM and DBM-DMSN groups. These results indicate that TPTD loading, particularly at the medium dose, enhanced osteogenic marker expression and promoted the progression of BMSCs toward a more mature osteogenic phenotype.

Osteogenic differentiation is a multi-stage, multi-gene co-regulated process in which BMSCs commit to the osteogenic lineage. RUNX2, as the core transcription factor of osteogenic differentiation, serves as an upstream master switch regulating the expression of multiple osteogenesis-related genes, including ALP and OCN [58]. In this study, the enhanced RUNX2 expression observed in the TPTD-loaded groups suggests that TPTD may promote the activation of osteogenic differentiation-related transcriptional programs. This discovery aligns with prior reports indicating that TPTD may upregulate RUNX2 expression through signaling pathways involving cAMP/PKA [59]. Accordingly, the cAMP/PKA-related pathway may contribute to the osteogenic effects of TPTD, although other signaling mechanisms may also be involved. Consistent with previous findings that intermittent TPTD boosts osteogenic differentiation of mesenchymal stem cells [60], the DBM-DMSN@TPTD-M group (10 nmol/L) demonstrated the strongest osteogenic response among all tested concentrations. OCN is a marker associated with osteoblast maturation and matrix mineralization [61], and its increased expression suggests the progression of BMSCs toward a more mature osteogenic phenotype.

In summary, the TPTD-loaded DBM-DMSN composite scaffolds promoted BMSC proliferation and osteogenic differentiation, among which the medium-dose group demonstrated the optimal effects in terms of ALP activity, RUNX2 expression, and OCN expression.

Orthopedic medicine continues to face a significant clinical hurdle in the form of critical-sized bone defects. These are bone defects that surpass the inherent ability of bone tissue to repair itself [2]. DBM preserves the three-dimensional extracellular matrix structure, collagen components, and mineralized microenvironment of native bone tissue, exhibiting favorable osteoconductive properties, and has therefore become an important research direction in bone repair materials in recent years [7]. However, the decellularization process removes a substantial portion of endogenous bioactive cytokines, resulting in limited osteoinductive capacity of DBM alone [62], which is insufficient to meet the demands of the complex regenerative environment in critical-sized bone defects.

Thus, how to further enhance the bioactivity of DBM while maintaining the biomimetic advantages of the native bone matrix has become a key direction in DBM functionalization research. In recent years, various functionalization strategies have been explored to improve the osteogenic performance of DBM. Parsaei et al. coated decellularized amniotic membrane gel onto DBM, significantly improving calvarial defect repair outcomes [63]. Li et al. incorporated decellularized Wharton’s jelly into DBM to construct a functionalized DBM scaffold, which promoted chondrogenic differentiation of BMSCs and subsequently activated endochondral ossification, achieving high-quality repair of osteochondral composite defects in a rabbit model [64]. In addition, ADM/DBM composite foam has been shown to enhance the mechanical properties and osteogenic capacity of DBM [65]. These studies collectively indicate that incorporating bioactive components to endow DBM with sustained osteogenic activity represents a promising strategy to enhance bone repair.

However, most of the aforementioned strategies rely on natural biomaterials to improve the local microenvironment, with less attention paid to the precise delivery and sustained release of active factors. In this study, the DBM-DMSN@TPTD composite scaffold was constructed to achieve an integration of material structure and biological function through the synergistic combination of native bone matrix, bioactive peptide, and nanocarrier system. Specifically, DBM provides a native extracellular matrix microenvironment that supports cell adhesion, migration, and proliferation. DMSNs serve as an effective local delivery system for TPTD, enabling sustained release and favorable drug release kinetics for bone repair applications [29,66].

In the present study, TPTD exhibited a cumulative release of approximately 83.23% over 16 days, beginning with a rapid phase and followed by sustained drug release. This release profile covers the critical time window of early cell recruitment and osteogenic differentiation during bone repair, providing favorable conditions for sustained osteogenic effects. Compared with previously reported DBM functionalization strategies, the most distinctive feature of our approach lies in the introduction of DMSN to construct a local sustained-release system, rather than relying solely on natural biomaterials to modify the microenvironment. This in situ delivery strategy enhances the local bioavailability of TPTD and minimizes systemic side effects. Although this composite scaffold combines the advantages of a native biomimetic microenvironment and controlled drug delivery, its full potential remains to be validated in vivo. Nonetheless, these in vitro findings offer a foundation for the subsequent advancement of DBM-DMSN@TPTD scaffolds as bone repair materials with sustained biological activity.

Several limitations of the present study should be noted. First, this work was conducted exclusively in vitro, and the osteogenic efficacy of the composite scaffold in the complex in vivo microenvironment remains to be validated in critical-sized bone defect animal models. Second, although ALP staining and RUNX2/OCN expression provided preliminary evidence for osteogenic differentiation, late-stage mineralization assays, such as Alizarin Red staining, were not included in the present study. Future studies will incorporate mineralization assays and additional osteogenic markers to further confirm the osteogenic potential of the scaffold. In addition, the long-term degradation behavior of the scaffold and the time-dependent retention and interfacial stability of the DMSN coating were not systematically evaluated in the present study. These factors may influence sustained TPTD release, scaffold integrity, and long-term biological performance. Previous studies have shown that mesoporous silica particles coated onto three-dimensional bioactive glass scaffolds remained largely attached to the scaffold surface after immersion under physiologically relevant conditions for up to 10 days, suggesting favorable short-term coating retention [67]. However, the coating strategy and substrate used in that study differed from those of the present DBM-DMSN system. DBM is mainly composed of a collagen-based extracellular matrix, and its gradual enzymatic degradation and remodeling are generally considered part of the normal tissue-regeneration process. The preserved extracellular matrix architecture of decellularized bone scaffolds can provide structural and biochemical cues that support cell adhesion and osteogenic differentiation [68]. Similarly, silica-based mesoporous materials may undergo hydrolytic dissolution of the siloxane network under physiological conditions [69]. Soluble silicon species released during silica degradation have also been reported to exhibit good cytocompatibility and to support osteogenic activity at appropriate concentrations [70]. Previous studies have reported favorable cytocompatibility of mesoporous silica materials under appropriate conditions, including good cell viability after exposure to dendritic mesoporous silica nanoparticles and MCM-41-type mesoporous silica nanoparticles [71,72]. These findings are consistent with our observation that DMSN modification did not compromise scaffold cytocompatibility. However, the dose-dependent toxicity of free DMSN was not evaluated in the present study and should be investigated in future work. Nevertheless, the cytocompatibility of free DMSN cannot be directly extrapolated to the scaffold-bound and aminated DMSN used in this study, because the biological response may depend on particle concentration, surface functionalization, aggregation state, and the surrounding DBM matrix. In addition, excessive or rapid scaffold degradation, DMSN detachment, or redistribution of the coating could compromise scaffold integrity and alter the release kinetics of TPTD. Therefore, future studies should systematically investigate DBM degradation, DMSN coating retention and distribution, local silicon release, in vivo TPTD release kinetics, and biosafety, as well as their relationships with bone regeneration and osseointegration.

## 4. Conclusions

We successfully constructed a DBM-DMSN@TPTD composite scaffold. The scaffold was prepared by decellularizing bovine cancellous bone via a combined freeze–thaw and chemical detergent method to obtain DBM with low immunogenicity, followed by grafting of DMSN onto the DBM surface through EDC/NHS crosslinking. Leveraging the high specific surface area and large pore size of DMSN, the scaffold achieved efficient loading and sustained release of TPTD. In vitro experiments confirmed the good biocompatibility of the composite scaffold. Upon TPTD loading, the scaffold significantly promoted BMSC proliferation and osteogenic differentiation. Among the tested concentrations, the medium-dose group (10 nmol/L) demonstrated the optimal performance in terms of ALP activity, RUNX2 expression, and OCN expression. This composite scaffold may warrant further investigation as a potential strategy for bone defect repair. Nevertheless, its efficacy in repairing critical-sized bone defects requires validation in appropriate animal models.

## Data Availability

Data will be made available on request.
